# Food Insecurity, Dietary Diversity, and Coping Strategies in Jordan during the COVID-19 Pandemic: A Cross-Sectional Study

**DOI:** 10.3390/nu14112252

**Published:** 2022-05-27

**Authors:** Amin N. Olaimat, Islam K. Alshami, Huda Al Hourani, Wafaa Sarhan, Murad Al-Holy, Mahmoud Abughoush, Narmeen Jamal Al-Awwad, Maha Hoteit, Ayoub Al-Jawaldeh

**Affiliations:** 1 Department of Clinical Nutrition and Dietetics, Faculty of Applied Medical Sciences, The Hashemite University, Zarqa 13133, Jordan; islamk@hu.edu.jo (I.K.A.); hhourani@hu.edu.jo (H.A.H.); murad@hu.edu.jo (M.A.-H.); mahmoud.abughoush@aau.ac.ae (M.A.); narmeen@hu.edu.jo (N.J.A.-A.); 2 Department of Nutrition and Food Technology, Faculty of Agriculture, Jordan University of Science and Technology, Irbid 22110, Jordan; wbsarhan17@agr.just.edu.jo; 3 Science of Nutrition and Dietetics Program, College of Pharmacy, Al Ain University, Abu Dhabi 64141, United Arab Emirates; 4 Faculty of Public Health, Lebanese University, Beirut 6573, Lebanon; m.hoteit@ul.edu.lb; 5 PHENOL Research Group (Public HEalth Nutrition prOgram Lebanon), Faculty of Public Health, Lebanese University, Beirut 6573, Lebanon; 6 Lebanese University Nutrition Surveillance Center (LUNSC), Lebanese Food Drugs and Chemical Administrations, Lebanese University, Beirut 6573, Lebanon; 7 Regional Office for the Eastern Mediterranean, World Health Organization, Cairo 7608, Egypt; aljawaldeha@who.int

**Keywords:** food security, food consumption, food-based coping strategies, COVID-19, Jordan

## Abstract

The 2019 coronavirus disease (COVID-19) is an emerging respiratory infection with severe impacts on the nutritional status of the worldwide population. This cross-sectional study was conducted to assess the food insecurity, dietary diversity, and food-related coping strategies in Jordan during the pandemic using an online, self-administered questionnaire. Among the 740 adults who completed the survey, the prevalence of food security was 84.1%, whereas 2% and 13.9% were moderately and severely food-insecure, respectively. The determinants of food insecurity were educational level, monthly income, marital status, availability of health insurance, and type of residence. In addition, food insecurity was significantly higher among the participants who consumed two or fewer meals per day (*p* = 0.015). Moreover, an acceptable food consumption score was shown among 76.2% of the participants, and the remaining participants were at borderline (14.1%) or had poor scores (9.7%), with a significant association between food insecurity and food consumption scores (*p* < 0.001). The food-related coping strategies studied were significantly associated with food insecurity at both levels (*p* < 0.001) and were more evident in the severely food-insecure group. These findings highlight the adverse effects of COVID-19 restrictions on nutritional status, especially among food-insecure households, which might reduce food accessibility due to economic difficulties.

## 1. Introduction

In December 2019, COVID-19, a disease caused by the SARS-CoV-2 virus, was discovered, which can lead to mild–moderate respiratory symptoms [1,2]. In most symptomatic infected people, the symptoms start within less than a week and include fever, cough, fatigue, loss of taste or smell, headache, dizziness, and generalized weakness [3,4]. Generally, COVID-19 patients can recover without special medical treatment; however, older adults and groups with certain medical conditions such as respiratory diseases, cardiovascular diseases, cancer, and diabetes may develop severe symptoms and require hospitalization [5]. According to WHO statistics as of 22 March 2022, the worldwide confirmed cases of COVID-19 are more than 480 million, and include more than 6.1 million deaths. There have been more than 1.7 million confirmed cases in Jordan, with more than 14,000 deaths by 22 March 2022 [6]. Since the early stages of the COVID-19 pandemic, the Ministry of Health in Jordan has encouraged people to follow the WHO recommendations to prevent the infection, such as receiving the COVID-19 vaccination, maintaining physical distancing for at least 1 m, wearing masks properly, frequent handwashing with soap and water for 20 s, and avoiding handshaking and touching the eyes, nose, and mouth [5,7]. Further, the Jordanian government has implemented several strategies to ameliorate the virus spread, such as a curfew in mid-March 2020, limitations in international travel and social events, and starting distance online learning [8,9,10]. Despite the beneficial effects of these strict regulations regarding disease control, many negative consequences on people’s lives have occurred [9]. The psychological impacts of COVID-19 and the associated restrictions are well-studied topics. For example, a systematic review reported a worsening in psychological health and scores of anxieties and depressive symptoms among the general population compared to the pre-COVID-19 period [11]. In Jordan, a cross-sectional study reported that 69.5% of the studied sample suffered from severe psychological distress [9]. In addition to the psychological effects, the Jordanian population’s eating patterns and overall health status were significantly affected by the lockdown during March and April 2020. Al-Domi et al. [8] found associations between the extended quarantine and a higher intake of macronutrients, main meals, and snacks between meals. In addition, a significant increase in smoking and body weight was reported. Furthermore, the effect of the COVID-19 pandemic on the global economy has become evident due to its significant impact on many sectors such as tourism, medicines, and manufactured products [12,13]. Low-income countries with limited resources were more exposed to economic consequences [9]. Moreover, the US reported an increase in its unemployment rate from 4.4% in March 2020 to 14.7% in May 2020 [14]. The association between household income and food security is strong [15]. Unfortunately, it is expected that over half a billion people may have become poor due to pandemic-related restrictions, which highlights the food insecurity risk [13]. The effect of COVID-19 on food security was studied in the US, and it was estimated that 54 million people would be food-insecure in 2020, which is around 17 million higher compared to 2018 [16]. Food insecurity is a situation where households have limited consistent access to enough, safe, and nutritious food to live an active and healthy life. Food insecurity is classified as mild, moderate, and severe [17]. Indeed, COVID-19 severely threatened the following essential aspects of food security: food availability, food access, food utilization, and food stability [13]. These effects occur mainly due to the pandemic’s disruptions on agricultural production, supply chains, and trade, in addition to food choices, i.e., it is suggested that there was a shift toward starchy food consumption, causing an increase in calories but a decrease in micronutrient intake [18]. Therefore, food insecurity has an adverse impact on health status, with consequences such as obesity, diabetes, peripheral insulin resistance, hypertension, hyperlipidemia, and nutrient deficiencies, in addition to its effect on mental and social status, especially among children and older adults [19,20]. Thus, the periodic investigation of the food security status of the worldwide population is critical, and is becoming increasingly important in the context of COVID-19. To the best of our knowledge, this is the first study evaluating food insecurity status, food consumption patterns, and food-related coping strategies among Jordanian households during the COVID-19 pandemic, post-quarantine. The specific aims of this study were therefore to: (1) assess the association of socio-demographic variables with food insecurity in Jordan during the COVID-19 pandemic, (2) assess the weekly food group consumption among Jordanian households and its association with food security status during the COVID-19 pandemic, and (3) assess the food-based coping strategies among Jordanian households and their association with food security status during the COVID-19 pandemic. 

## 2. Materials and Methods

### 2.1. Study Design 

This was a cross-sectional study conducted in Jordan. The validated Arab Food Security Scale survey questionnaire [21] was used to collect the data (Supplementary). The data were collected using a self-administered online survey. A non-probability sample of social media users on Facebook, Messenger, and WhatsApp was recruited by conducting an advertisement linked to Facebook and WhatsApp. Recruitment occurred from 28 September to 8 December 2021, and the advertisement targeted adults aged ≥ 18 years residing in Jordan.

### 2.2. Ethical Approval 

The study was proved by the Institutional Review Board at the Hashemite University (IRB No. 8/13/2020/2021).

### 2.3. Survey Tool

The questionnaire involved an introduction showing the study’s objectives, which highlighted that the answers of participants would be treated confidentially. The participants were not paid for their involvement in this study and it was entirely voluntary. The completion of the online survey took approximately 8–10 min. The data were presented as multiple-choice questions in a structured survey questionnaire. The first section determined participants’ socio-demographic variables, including gender, marital status, age, education level, employment status, number of children, urban/rural residence, number of household members, and monthly household income. The next sections assessed Jordan’s food security, food consumption, and coping strategies during the COVID-19 pandemic. 

### 2.4. Sample Size

The minimum sample size needed to estimate the prevalence of food insecurity within a margin of error of 5% at a significance level of 5% and a power of 80% was 384. The sample size was increased to have a higher power.

### 2.5. Data Classification

Food insecurity measures the severity of food insecurity based on people’s responses to questions about the constraints on their ability to obtain adequate food. It was assessed using the eight-item scale by adopting the Arabic version of The Food Insecurity Experience Scale (FIES) that was developed by the Food and Agriculture Organization (FAO) [22]. Participants were asked about food insecurity concerns over the past six months—the scores in this survey range from 0 to 1. Respondents answered yes/no to the eight questions, and the responses were aggregated to give raw scores ranging from 0 to 8. Food insecurity (FI) was divided into three categories, including: (1) food-secure (FS) with raw scores = 0–3; (2) moderate food insecurity (MFI), with raw scores = 4–6; and (3) severe food insecurity (SFI), with raw scores = 7–8 [23]. 

The Food Consumption Score (FCS) was based on a household’s frequency of consumption of various food groups over the past seven days. A household food consumption score was calculated by multiplying each food group frequency by each food group weight (Table 1), and then summing these scores into one composite score [24].

A household score was compared with pre-established thresholds that indicated that household’s food consumption status. The World Food Program (WFP) finds the following thresholds applicable in a wide range of situations: poor food consumption: 0 to 21; borderline food consumption: 21.5 to 35; and acceptable food consumption: > 35. A household score could have a maximum value of 112, which implied that each food group was consumed every day for the last seven days. 

### 2.6. Statistical Analysis

All statistical analyses were conducted using the Statistical Package for Social Sciences (SPSS) software (IBM Corp., released 2013; IBM SPSS Statistics for Windows, Version 22.0, Armonk, NY, USA: IBM Corp). The chi-square test was used for the categorical variables. Multinomial logistic regression was used in estimating the association between the selected household characteristics and food security status. In this case, households were classified as moderately food-insecure and severely food-insecure. A *p*-value of < 0.05 was considered statistically significant.

## 3. Results

### 3.1. Socio-Demographic Characteristics

A total of 740 Jordanian adults participated in this study; of them, 160 (21.6%) were males and 580 (78.4%) females. Their socio-demographic characteristics classified according to food security status are listed in Table 2. The majority of the participants were younger than 50 years (92.7%), had a diploma or bachelor’s at an educational level (67.8%), lived in the city (85.4%), lived in their own residence (70.7%), and had health insurance (66.4%). More than half of our study population was single (55%) and had a family with 4–7 members (52.4%). Further, about 45% of the participants had a household income of between JOD 500 and 1000 (USD 700–1400).

### 3.2. Household Food Security Status

The prevalence of food security among the study population was 84.1%, while 2% were classified as moderately food-insecure and about 14% as severely food-insecure, as shown in Table 2. Associations between socio–demographic characteristics and food insecurity are also presented in Table 2. A significant association was found between the presence of food insecurity and having a low educational level, being divorced or widowed, having more than three children, having a maximum of one employed family member, living in a rented residence, not having health insurance, and an overall household income of lower than JOD 500 (*p* < 0.05).

### 3.3. Determinants of Household Food Insecurity 

As many socio-demographic factors are significantly associated with food security, a multinomial logistic regression analysis was performed to evaluate the strength of the relationship between these variables and the risk of being food-insecure at both levels, moderate or severe, as shown in Table 3. Educational level, marital status, and income level were all determinants of being severely food-insecure, while the unavailability of health insurance was the determinant that could lead to being moderately food-insecure. The results showed that the participants with a low educational level were more likely to be severely insecure by about seven times (95% CI = 1.104–3.978; *p* < 0.001) for those with secondary school or less and two times (95% CI = 3.401–15.881; *p* < 0.05) for those with a diploma or bachelor’s degree when compared with postgraduate participants. Participants with a monthly income of less than JOD 500 were about seven times more likely to be severely food-insecure when compared with participants achieving more than JOD 1000 (95% CI =3.336–13.651; *p* < 0.001). Single participants less likely to be severely food-insecure, with an OR = 0.219 (95% CI = 0.077–0.622; *p* < 0.05). 

Further, the availability of health insurance was found to be a helpful factor that protected participants from being moderately food-insecure (OR (95% CI) = 0.167 (0.053–0.532); *p* < 0.05). Moreover, participants who were living in their own residences tended to be at a lower risk of being food-insecure at both levels (moderately food-insecure, OR = 0.308 (95% CI = 0.110–0.863; *p* < 0.05), and severely food-insecure, OR = 0.420 (95% CI = 0.274–0.643; *p* < 0.05)) (Table 3).

### 3.4. Weekly Food Group Consumption 

Approximately 63% of the participants consumed two or fewer meals daily, and a higher proportion of them consumed almost all of the food groups in a frequency of 3 days or fewer per week, as shown in Table 4. Food insecurity was significantly higher among the participants who consumed two or fewer meals (3% and 15.3% vs. 0.4% and 11.6% (*p* = 0.015) for moderately and severely food-insecure; respectively) compared with those who consumed more meals. Furthermore, a higher prevalence of food insecurity was significantly shown for participants who reported that they consumed meals less than usual (about 5% moderate food insecurity and 29% severe food insecurity) (*p* < 0.001). 

With respect to food group weekly consumption, all the main food groups were significantly associated with food security status. Participants who consumed cereals, vegetables, fruits, eggs, legumes and nuts, milk and dairy products, oil and fat, sugar, spices and condiments, and meat and poultry at a frequency of 3 days or fewer per week tended to be more food-insecure than participants who consumed these items more during the week (Table 4).

### 3.5. Food Consumption Scores

**Figure 1:**
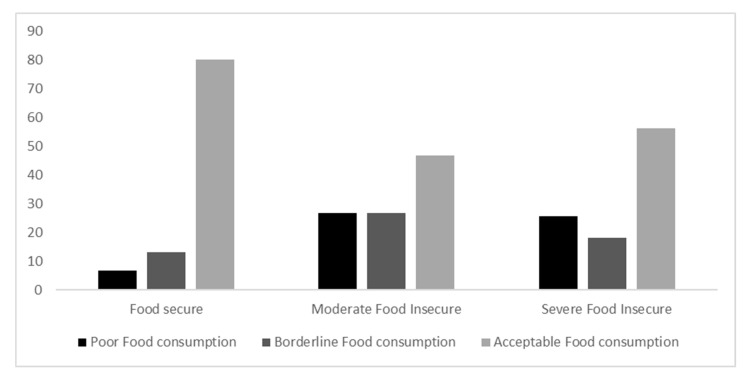
Food consumption scores among the participants according to food security status.

The weekly food group consumption was translated into food consumption scores and classified into three groups: poor (9.7%), borderline (14.1), and the remaining (76.2%) were classified as consuming food at an acceptable level. There was a strong significant association between food insecurity, at both levels of severity, and the food consumption scores, as shown in Figure 1 (*p* < 0.001). The associations were stronger with the more severe range of food insecurity. These findings were also reflected in poor food consumption scores, which were significantly lower among food-secure households (Figure 1).

### 3.6. Food-Based Coping Strategies during the Last Week

During the last week of response, more than 96% of the participants were following food-based coping strategies for three days per week or less. Participants reported that they were eating cheaper food, borrowed food, and fewer meals or small amounts of food, and there were even adults eating less food to spare it for their children (Table 5). The associations between food-based coping strategies and food security status are presented in Table 5. Almost all the coping strategies were significantly associated with food insecurity at both levels (*p* < 0.001), but this association was evident in the severely insecure group. Participants with severe food insecurity tended to practice these coping strategies at least four days during the week, and the results revealed that 45.5% of those participants ate cheaper food, 25% borrowed food, 36.4% ate fewer meals, 39.1% ate a small amount of food, and 13.9 % were instances where only adults ate less, compared to food-secure participants. 

In a separate analysis for non-food-based coping strategies during the last 30 days, approximately 34% of the participants did not use any strategy to meet basic food needs while 28% spent money savings to meet basic food needs, and 16% spent less money on other needs such as education and health. The rest of the participants used different strategies, such as buying food on credit, borrowing money, or doing any labor for food (results not shown).

### 3.7. Food Quality and Quantity

**Figure 2:**
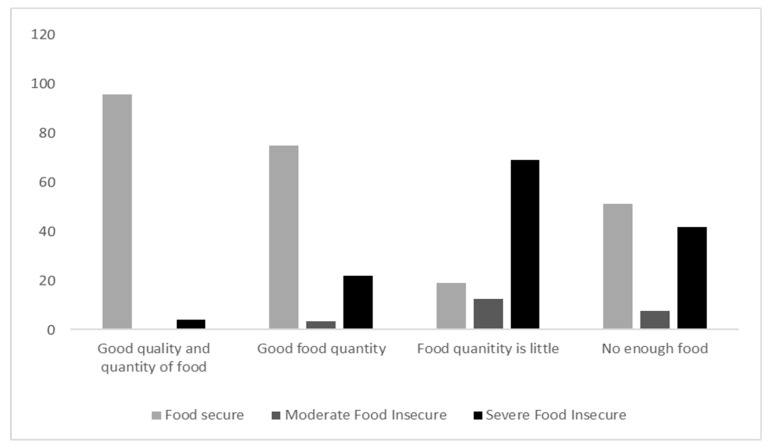
Quality and quantity of consumed foods during the COVID-19 pandemic.

The food quality and quantity were affected by the level of food insecurity. Almost 95% of the food-secure respondents had good qualities and quantities of food, while 68% of the severe food-insecure participants had smaller quantities of food and 21% had a good quality of food (Figure 2).

## 4. Discussion

This cross-sectional study found that most of the studied population was food-secure (84.1%), whereas 2% were moderately food-insecure and 13.9% were severely food-insecure. These results differ from a previous study conducted in Jordan during the COVID-19 quarantine (n = 3129), which found that the prevalence of food security was 40.7%, moderate food insecurity was 36.1%, and severe food insecurity was 23.1% [25]. Compared with Elsahoryi et al.’s results [25], the improvement in food security status is expected due to the demise of some negative factors associated with the quarantine, such as the lockdowns and movement difficulty; thus, the limitations in food accessibility were reduced. In the present study, there is a significant impact of educational level on food security wherein participants with low educational levels were more likely to be severely food-insecure. This association is consistent with several past studies conducted in both rural and urban areas [26,27,28].

Education plays an important role in employment opportunities, working efficiency, accessing information about health and nutrition, increasing income, and diversity, all of which improve household food supply [29,30]. Our results showed that being divorced or widowed is another significant factor associated with food insecurity, and that being single decreases the risk of severe food insecurity. The association between marital status and household food security varies in the literature. For example, studies of households in Kenya and Nigeria (n = 209 and 180, respectively) reported that married respondents were more likely to be food-secure due to the contribution of both the husband and wife to family income, which in turn improved food security status [31,32]. The average monthly wage income of professional employees in Jordan in 2019 was JOD 692 (USD 970) [33]. On the contrary, a cross-sectional study in Iran (n = 1385) documented that married respondents were less likely to be food-secure [34]. A similar finding was reported in a study of 100 households in Ghana, and this could be attributed to the smaller number of family members, which may reduce a family’s economic burden [35]. The present study found that having more than three children negatively impacted food security. The larger family size increased the competition for the available resources. A higher number of children (an inactive age group) was associated with less production and more consumption [36].

Furthermore, our results found a significant association between food insecurity and a maximum of one working member in the household. During the COVID-19 pandemic, 7% of Jordanians lost their jobs and the unemployment rate reached more than 23% in 2021 compared to approximately 19% in 2019 [37,38]. Loopstra and Tarasuk [39] reported that a change in the number of full-time employed household members was associated with the severity of food insecurity independent of the change in household income, and they explained this association by improving access to dental insurance and prescription drugs, which enabled families to save money for food. This may explain our results regarding the association between having health insurance and food security, where the unavailability of health insurance was a determinant of moderate food insecurity. Further, the relationship between the number of employed household members and food security may be related to the large effect of the number of employees on family income, as employment is one of the main sources of total income [40]. Indeed, the COVID-19 pandemic had a critical influence on households’ incomes in different countries. For example, a study of 1821 Nigerian households showed that 79% reported a decline in household income during the pandemic restrictions [41]. The current study found that monthly income level is one of the determinants of severe food insecurity. Participants with a monthly income of less than JOD 500 were about seven times more likely to be severely food-insecure than participants with incomes of more than JOD 1000. In line with our results, Elsahoryi et al.’s cross-sectional study in Jordan reported that participants with a per capita monthly income of below the poverty line were more susceptible to moderate and severe food insecurity during the quarantine period in 2020 [25]. Similarly, the association between low-income levels and food insecurity has been documented in several countries such as Turkey [42], South Africa [43], Sudan [44], Bangladesh [45], Nigeria [41], and Ethiopia [46].

Regarding the type of residence, this study found that living in one’s own residence decreases the likelihood of moderate and severe food insecurity compared to living in a rented residence. Studies have reported that housing costs reduce food access and, in turn, disturb the food security status of low-income families [47]. Further, and consistent with our results, people who lived in rented houses were at a higher risk of severe food insecurity than people who lived in owned houses during the COVID-19 quarantine in Jordan [25].

The current study indicates that around 63% of the participants consumed two or fewer meals per day, and this frequency was reported “as usual” by 87% of the participants. The consumption of 1–2 meals per day was higher before the COVID-19 pandemic and found to be 85% [48]. Only 2.3% of the participants in the current study reported an increase in the number of meals than usual. Higher percentages were reported in an earlier study of 4473 participants in Jordan during the lockdown, where 69.4%, 89.9%, and 54% of the sample reported an increase in breakfast, lunch, and dinner numbers, respectively [8]. The effect of the COVID-19 pandemic on the number of daily meals consumed was studied in different countries; a cross-sectional study of 2970 adults residing in the Middle East and North Africa regions found that 6.2% were consuming five or more meals per day during the COVID-19 pandemic compared to 2.2% pre-COVID-19, and 45.1% were skipping meals during the pandemic compared to 64.4% before [49]. Likewise, a study of 1012 adult residents of the United Arab Emirates compared the number of daily meals consumed before lockdown and during the lockdown and reported an increase in the consumption of 3-4 meals per day from 51.5% to 56.5%, and an increase of ≥ 5 meals per day consumption from 2.1% to 7% [50]. Further, a study of 866 university students in Turkey found that 23.0% of the participants increased the number of their daily meals during the pandemic compared to before [51]. Similarly, a study of 1932 Italians showed that 46.1% of the respondents consumed more than usual during the lockdown [52]. Cross-sectional studies from other countries also reported more meals consumed during the lockdown than before, such as India [53] and Saudi Arabia [54]. These findings show how the pandemic and its associated lockdowns have affected dietary patterns, which could be due to a variety of factors, including stress, anxiety, boredom, and the longer time available for eating during quarantine compared to working and studying outside the home, and food security status [55,56,57]. Certainly, our results showed that moderate and severe food insecurity occurs at a significantly higher prevalence among participants who consumed two or fewer meals and reported a “less than usual” number of meals compared to those who consumed more than two meals and reported a “more than usual” or “as usual” number of meals. This is in agreement with a study in Saudi Arabia (n = 879), which indicated that changes in the number of meals per day during the COVID-19 curfew were frequent among participants with severe food insecurity. These changes occurred mainly due to food unavailability [58].

In addition, the present study showed that most participants consumed almost all the main food groups 3 days or less per week. A significant association was shown between food insecurity and a frequency of 3 days or fewer per week consumption of cereals, vegetables, fruits, eggs, legumes and nuts, milk and dairy products, oil and fat, sugar, spices and condiments, meat, and poultry. When translated to food consumption scores, the current results report that 76.2% of the participants were within acceptable levels, 14.1% were classified as borderline, and 9.7% were within poor consumption levels, with a significant association between food insecurity and food consumption scores. Further, consuming good quality and quantities of food was only shown in 4.3% of food-insecure respondents. The food consumption score was studied among other countries in the region and found to have worsened during the COVID-19 pandemic; for example, a cross-sectional study of 2282 Lebanese participants reported a decrease in the average food consumption score by 4.6% during the lockdown [59]. These results could be due to the decrease in households’ food purchasing power during the pandemic, which was more prevalent among food-insecure households, leading to a shortage of food access and thus decreasing food quantity and quality. However, Elsahoryi et al.’s study during the quarantine in Jordan showed no associations between weekly food group intake and food security status, except for the carbohydrate (higher intake among the severe food-insecure group) and meat groups (higher intake among the food-secure group) [25]. This further highlights the influence of economic status on the variety of foods consumed, as the price of food items from the carbohydrates group is lower than the meat, poultry, and fish group.

Most of our study respondents (96%) reported using food-based coping strategies three days per week or less. Significant associations were found between food insecurity and eating cheaper foods, eating fewer meals or small amounts of food, and adults eating less food to spare it for their children. However, this association was evident in the severely insecure group as they followed these coping strategies at least four days during the week. The world food program reported an increase in the percentage of Jordanian households following consumption-based coping strategies from 23% in 2014 to 55% during the pandemic, with 25% reducing the number of meals/day and 10% relying on friends and family for help with food shortages. This occurred mainly due to a loss of employment and income [38]. Consistent with these results, the data from a multi-country survey of 1075 participants showed that 49.2% relied on less-preferred food during the pandemic, 30.3% reduced portion size, and 25.7% reduced the number of meals. In addition, they used other coping strategies, such as restricting food for adults (18.7%) and borrowing food (17%) [60]. Furthermore, the association between food insecurity and food-based coping strategies was studied in food-insecure households in Ethiopia (n = 6671), and it showed that most households responded to food insecurity by managing food left in their homes. Decreasing the amount and frequency of meals was the most prevalent coping strategy (55.96%), followed by borrowing food and money (38.11%) [61]. Similarly, a study of Vermont households (n = 3219) revealed that food-insecure participants followed food-related coping strategies significantly more than food-secure households. This result applied to families who were food-insecure before the pandemic, as well as families who became food-insecure after the pandemic [62]. It is obvious that implementing food-related coping strategies is common for dealing with food insufficiency, which highlights the seriousness of the problem, especially among food-insecure households in the context of the COVID-19 pandemic.

Compared with the results presented in the current study, the previous studies in Jordan showed a lower prevalence of food insecurity before the COVID-19 pandemic. In 2013–2014, a study of 24,740 households reported that 93.8% of the sample were food-secure and 0.5% were food-insecure, whereas 5.7% were vulnerable to food insecurity [63]. Earlier, in 2010, the prevalence of food security was higher (97.5%) among the studied households (n = 13,888), while food insecurity was found in 0.3%, and 2.1% were vulnerable to food insecurity [64]. These results may reflect a negative influence of the COVID-19 pandemic on food security status. Unfortunately, this effect could not be confirmed due to the lack of studies on food security status among the general population in Jordan shortly before the pandemic.

However, there are other studies of pre-pandemic food security status focused on specific groups such as women, vulnerable Jordanian communities, and children. A study of women in Northern Jordan (n = 500) found that 67.6% of the interviewed sample were from food-secure households, and 32.4% were from food-insecure households [65]. Further, a survey of 4241 vulnerable Jordanian households found a prevalence of food security (30%), risk of food insecurity (59%), and food insecurity (11%) [66]. A lower prevalence of food insecurity was found in 2008 by WFP [66]. Among 3000 vulnerable Jordanian households, 72.7% were food-secure, 19.2% were at risk of food insecurity, and 7.9% were food-insecure. Additionally, food security status was assessed among children (12–16 years) in Northern Jordan (n = 679), and it showed that the food security among children was 78%, while the mild and severe food insecurity were 15.3% and 6.8%, respectively [67]. Moreover, several studies have focused on the food security status of refugees [68,69,70,71]. The results of the current study could not be compared with the results of these studies due to the variety of the studied groups.

The study findings indicate the need for scaling up the implementation of national strategies to achieve food security by 2030 through addressing, holistically, all aspects of food security and adopting appropriate and resilient food systems, especially with the impact of the ongoing conflict in Ukraine, which is heightening concerns about the impact on food security worldwide. We do recommend that the decision-makers and health-promotion professionals and institutions in Jordan plan interventional programs to reduce the consequences and magnitude of food insecurity, with an increased focus on social protection, care and nutrition services, and governance in Jordan, aiming for scaling up food security and food systems interventions, including the following recommendations:

1. Ensure the availability of food at the national, household, and individual levels, with the following sub-objectives:
  A Achievement of the maximum potential of local food production
  B Provision of a sufficient and stable supply of imported items
  C Improvement of regional collaboration and integration of the different aspects of food security
  D Reduction in food loss and waste, and enhanced food safety
2. Optimize the utilization and stability of food, with the following sub-objectives:
  A Improvement of food quality for all people in Jordan

## 5. Study Strengths and Limitations

This study updates the data about food security status, its determinants, its effect on food consumption patterns, and the related household coping strategies. It is important to study these variables periodically, especially in low- and middle-income countries. The importance has increased considering the spread of COVID-19 and its economic consequences during the lockdown and beyond. In the present study, one of the main limitations was the young median age of the respondents, which may have occurred due to the use of social media as the distribution tool. Besides, online self-reporting questionnaires may lead to bias due to over- or under-estimation of the questions. However, the sample size, which was almost twice the size needed, is one of the main strengths of the present study, in addition to the fact that the participants in the current study were recruited from several regions in Jordan, and a validated questionnaire for measuring food security status was used.

## 6. Conclusions

Although the overall food security prevalence seems to have improved after the lockdown, around 16% of the participants are still food-insecure. Food insecurity was significantly associated with low educational level, being divorced or widowed, having large family size, a low number of the household members being employed, not having health insurance, and having a rented residence. Besides, most of the sample reported two or fewer meals consumed per day and a frequency of three days or less intake of almost all the main food groups per week. As expected, food insecurity was significantly higher among the participants who skipped meals and food groups, reported a “less than usual” number of meals, and had lower food consumption scores. Moreover, significant associations were found between food insecurity and food-based coping strategies such as eating cheaper foods, eating fewer meals or small amounts of food, and adults eating less food to spare it for their children. These results are consistent with results from numerous studies conducted worldwide, and they further underscore the impact of the COVID-19 pandemic on food consumption patterns, especially among food-insecure households, which may occur due to the loss of food accessibility, and hence decrease the quantity and quality of food consumption. These findings suggest severe impacts on people’s nutritional, mental, and social health; therefore, there is a need for implementing national strategies to increase food access, such as stabilizing the prices of food products, providing employment opportunities, supporting small businesses, and increasing health and nutrition awareness. It is recommended to conduct further studies among other groups susceptible to food insecurity, such as people with chronic diseases, children, and refugees.

## Figures and Tables

**Table 1:** Food groups and their particular weights.

Food Group	Food Items (Examples)	Weight
Main staples	Bread, rice, macaroni, cereals	2
Pulses	Broad beans, beans, chickpeas	3
Vegetables	All types of vegetables	1
Fruits	All types of vegetables	1
Meat/Fish	Beef, lamb, poultry, eggs, fish, organ meat	4
Milk	Milk, yogurt, cheese, labneh	4
Sugar	Sugar, sugar product, honey	0.5
Oil	Vegetable oils, butter, ghee	0.5
Condiments	Spices	0

**Table 2:** Socio-demographic characteristics of participants with data on household food insecurity during the COVID-19 pandemic.

Variables		Total	Food Security Status			*p*-Value
			Food-Secure 622 (84.1)	Moderate Food Insecurity 15 (2.0)	Severe Food Insecurity 103 (13.9)	
Gender	Male	160 (21.6)	133 (83.1)	3 (1.9)	24 (15.0)	0.898
	Female	580 (78.4)	489 (84.3)	12 (2.1)	79 (13.6)	
Age	18–49 years	686 (92.7)	576 (84.0)	12 (1.7)	98 (14.3)	0.106
	50 years and more	54 (7.3)	46 (85.2)	3 (5.6)	5 (9.3)	
Educational level	Secondary school or less	68 (9.2)	43 (63.2)	1 (1.5)	24 (35.3)	<0.001
	Diploma or bachelor	502 (67.8)	421 (83.9)	14 (2.8)	67 (13.3)	
	Postgraduate	170 (23.0)	158 (92.9)	0 (0.0)	12 (7.1)	
Marital status	Single	407 (55.0)	360 (88.5)	4 (1.0)	43 (10.6)	0.001
	Married	315 (42.6)	251 (79.7)	10 (3.2)	54 (17.1)	
	Divorced or widowed	18 (2.4)	11 (61.1)	1 (5.6)	6 (33.3)	
Number of children	None	472 (63.8)	416 (88.1)	4 (0.8)	52 (11.0)	0.003
	Less than 3	109 (14.7)	86 (78.9)	4 (3.7)	19 (17.4)	
	3–5 children	137 (18.5)	103 (75.2)	6 (4.4)	28 (20.4)	
	More than 5 children	22 (3.0)	17 (77.3)	1 (4.5)	4 (18.2)	
Residence	City	632 (85.4)	531 (84.0)	15 (2.4)	86 (13.6)	0.239
	Village	108 (14.6)	91 (84.3)	0 (0.0)	17 (15.7)	
Job of the head of the family	Employee at private sector	268 (36.2)	229 (85.4)	4 (1.5)	35 (13.1)	0.624
	Employee at governmental sector	195 (26.4)	165 (84.6)	4 (2.1)	26 (13.3)	
	Free business	159 (21.5)	130 (81.8)	6 (3.8)	23 (14.5)	
	Unemployed	118 (15.9)	98 (83.1)	1 (0.8)	19 (16.1)	
Number of employees working in the family	0–1 person	387 (52.3)	312 (80.6)	7 (1.8)	68 (17.6)	0.023
	2–4 people	334 (45.1)	291 (87.1)	8 (2.4)	35 (10.5)	
	More than 4	19 (2.6)	19 (100.0)	0 (0.0)	0 (0.0)	
Type of residence	Own residence	523 (70.7)	460 (88.0)	7 (1.3)	56 (10.7)	<0.001
	Rented residence	217 (29.3)	162 (74.7)	8 (3.7)	47 (21.7)	
Monthly income	Less than JOD 500 *	218 (29.5)	153 (70.2)	6 (2.8)	59 (27.1)	<0.001
	JOD 501–1000	334 (45.1)	294 (88.0)	6 (1.8)	34 (10.2)	
	More than JOD 1000	188 (25.4)	175 (93.1)	3 (1.6)	10 (5.3)	
Number of family members	Less than 4	260 (35.1)	218 (83.8)	5 (3.3)	37 (14.2)	0.694
	4–7 members	388 (52.4)	331 (85.3)	7 (1.8)	50 (12.9)	
	More than 7	92 (12.4)	73 (79.3)	3 (3.3)	16 (17.4)	
Availability of health insurance	Yes	491 (66.4)	426 (86.8)	4 (0.8)	61 (12.4)	0.001
	No	249 (33.6)	196 (78.7)	11 (4.4)	42 (16.9)	

* JOD 1 = USD 1.4, the average income is JOD 500.

**Table 3:** Multinomial logistic regression analysis of food security status in Jordan during the COVID-19 pandemic.

Variables		Food Security Status	
		Moderate Food Insecurity	Severe Food Insecurity
Gender	Male	0.919 (0.256–3.306)	1.117 (0.681–1.833)
	Female	*Reference*	*Reference*
Age	18–49 years	0.319 (0.087–1.172)	1.565 (0.607–4.037)
	50 years and more	*Reference*	*Reference*
Educational level	Secondary school or less	ND	7.349 (3.401–15.881) **
	Diploma or bachelor	ND	2.095 (1.104–3.978) *
	Postgraduate	*Reference*	*Reference*
Marital status	Single	0.122 (0.013–1.185)	0.219 (0.077–0.622) *
	Married	0.438 (0.051–3.734)	0.394 (0.140–1.113)
	Divorced or widowed	*Reference*	*Reference*
Number of children	None	0.163 (0.017–1.542)	0.531 (0.172–1.639)
	Less than 3	0.791 (0.083–7.519)	0.939 (0.284–3.109)
	3–5 children	0.990 (0.112–8.745)	1.155 (0.360–3.710)
	More than 5 children	*Reference*	*Reference*
Residence	City	ND	0.867 (0.492–1.527)
	Village	*Reference*	*Reference*
The job of the head of the family	Employee at the private sector	1.712 (0.189–15.511)	0.788 (0.430–1.446)
	Employee at governmental sector	2.376 (0.262–21.560)	0.813 (0.428–1.545)
	Free business	4.523 (0.536–38.182)	0.913 (0.471–1.769)
	Unemployed	*Reference*	*Reference*
Type of residence	Own residence	0.308 (0.110–0.863) *	0.420 (0.274–0.643) **
	Rented residence	*Reference*	*Reference*
Monthly income	Less than JOD 500	2.288 (0.563–9.302)	6.748 (3.336–13.651) **
	JOD 501–1000	1.190 (0.294–4.820)	2.024 (0.976–4.197)
	More than JOD 1000	*Reference*	*Reference*
Number of family members	Less than 4	0.558 (0.130–2.393)	0.774 (0.407–1.474)
	4–7 members	0.515 (0.130–2.037)	0.689 (0.372–1.278)
	More than 7	*Reference*	*Reference*
Availability of health insurance	Yes	0.167 (0.053–0.532) *	0.668 (0.436–1.025)
	No	*Reference*	*Reference*

* *p* < 0.05, ** *p* < 0.001.

**Table 4:** Weekly food group consumption among the study population stratified by food security status.

Variables		Total	Food Security Status			*p*-Value
			Food-Secure	Moderate Food Insecurity	Severe Food Insecurity	
Number of meals per day (1 day before)	2 meals or less	465 (62.8)	380 (81.7)	14 (3.0)	71 (15.3)	0.015
	More than 2 meals	275 (37.2)	242 (88.0)	1 (0.4)	32 (11.6)	
The actual number of meals as reported	As usual	644 (87.0)	554 (86.0)	11 (1.7)	79 (12.3)	<0.001
	Less than usual	79 (10.7)	52 (65.8)	4 (5.1)	23 (29.1)	
	More than usual	17 (2.3)	16 (94.1)	0 (0.0)	1 (5.9)	
Food group consumption in the previous 7 days						
Cereals	3 days and fewer	306 (41.4)	240 (78.4)	9 (2.9)	57 (18.6)	0.002
	4 days and more	434 (58.6)	382 (88.0)	6 (1.4)	46 (10.6)	
White tubers	3 days and fewer	654 (88.4)	544 (83.2)	14 (2.1)	96 (14.7)	0.201
	4 days and more	86 (11.6)	78 (90.7)	1 (1.2)	7 (8.1)	
Vegetables	3 days and fewer	431 (58.2)	345 (80.0)	12 (2.8)	74 (17.2)	0.002
	4 days and more	309 (41.8)	277 (89.6)	3 (1.0)	29 (9.4)	
Fruits	3 days and fewer	453 (61.2)	354 (78.1)	13 (2.9)	86 (19.0)	<0.001
	4 days and more	287 (38.8)	268 (93.4)	2 (0.7)	17 (5.9)	
Eggs	3 days and fewer	591 (79.9)	487 (82.4)	15 (2.5)	89 (15.1)	0.024
	4 days and more	149 (20.1)	135 (90.6)	0 (0.0)	14 (9.4)	
Legumes and nuts	3 days and fewer	563 (76.1)	458 (81.3)	13 (2.3)	92 (16.3)	0.002
	4 days and more	177 (23.9)	164 (92.7)	2 (1.1)	11 (6.2)	
Milk and dairy products	3 days and fewer	473 (63.9)	378 (79.9)	13 (2.7)	82 (17.3)	<0.001
	4 days and more	267 (36.1)	244 (91.4)	2 (0.7)	21 (7.9)	
Oil and fat	3 days and fewer	415 (56.1)	330 (79.5)	12 (2.9)	73 (17.6)	0.001
	4 days and more	325 (43.9)	292 (89.8)	3 (0.9)	30 (9.2)	
Sugar	3 days and fewer	434 (58.6)	343 (79.0)	13 (3.0)	78 (18.0)	<0.001
	4 days and more	306 (41.4)	279 (91.2)	2 (0.7)	25 (8.2)	
Spices and condiments	3 days and fewer	341 (46.1)	260 (76.2)	12 (3.5)	69 (20.2)	<0.001
	4 days and more	399 (53.9)	362 (90.7)	3 (0.8)	34 (8.5)	
Meat and poultry	3 days and fewer	369 (49.9)	279 (75.6)	10 (2.7)	80 (21.7)	<0.001
	4 days and more	371 (50.1)	343 (92.5)	5 (1.3)	23 (6.2)	
Fish	3 days and fewer	706 (95.4)	593 (84.0)	14 (2.0)	99 (14.0)	0.873
	4 days and more	34 (4.6)	29 (85.3)	1 (2.9)	4 (11.8)	

**Table 5:** Food-based coping strategies in the previous seven days.

Variables		Total	Food Security Status			*p*-Value
			Food-Secure	Moderate Food Insecurity	Severe Food Insecurity	
Eating cheaper foods	3 days and fewer	718 (97.0)	610 (85.0)	15 (2.1)	93 (13.0)	<0.001
	4 days and more	22 (3.0)	12 (54.5)	0 (0.0)	10 (45.5)	
Borrowing food	3 days and fewer	728 (98.4)	614 (84.3)	14 (1.9)	100 (13.7)	0.142
	4 days and more	12 (1.6)	8 (66.7)	1 (8.3)	3 (25.0)	
Eating less meals to spare food for children	3 days and fewer	718 (97.0)	610 (85.0)	13 (1.8)	95 (13.2)	<0.001
	4 days and more	22 (3.0)	12 (54.5)	2 (9.1)	8 (36.4)	
Eating small amounts	3 days and fewer	717 (96.9)	611 (85.2)	12 (1.7)	94 (13.1)	<0.001
	4 days and more	23 (3.1)	11 (47.8)	3 (13.0)	9 (39.1)	
Adults only eat less to spare food for children	3 days and fewer	715 (96.6)	609 (85.2)	14 (2.0)	92 (12.9)	<0.001
	4 days and more	25 (3.4)	13 (52.0)	1 (4.0)	103 (13.9)	

## Data Availability

Not applicable.

## Supplementary Materials

The following are available online at https://www.mdpi.com/article/10.3390/nu14112252/s1, Survey of Food Security in Jordan during COVID-19 Pandemic.
