# Supplementary material for: Food Insecurity, Dietary Diversity, and Coping Strategies in Jordan during the COVID-19 Pandemic: A Cross-Sectional Study
Source: Nutrients. 2022 May 27;14(11):2252. doi: 10.3390/nu14112252 (PMC9182708; doi:10.3390/nu14112252)
Supplement: Supplementary file 1 [file nutrients-14-02252-s001.zip › nutrients-1711696-supplementary.pdf]

Survey of Food Security in Jordan during COVID-19 Pandemic

This survey aims to study Food Security in Jordan during COVID-19 Pandemic. Any information that you provide will be kept strictly confidential and will not be shown to other people. This is voluntary and you can choose not to answer any or all of the questions if you want; however, we hope that you will participate since your views are important.

Participant consent

Do you agree to participate in this study?

- ☐ Yes
- ☐ No

Participant information

1. Gender:

- ☐ Male
- ☐ Female

2. Age category:

- ☐ 18-49 years
- ☐ 50 years or more

3. Educational level:

- ☐ High school or less
- ☐ Diploma or bachelor's degree
- ☐ Postgraduate studies

4. Marital Status

- ☐ Single (never married)
- ☐ Married
- ☐ Divorced or widowed

5. Number of children

- None
- Less than 3
- 3- 5 children
- More than 5 children

6. Place of residence:

- City
- Village

7. Employment of household head

- Employee at private sector
- Employee at governmental sector
- Free business
- Unemployed

8. Number of employees working in the family

- 0-1 person
- 2 - 4 persons
- More than 4 persons

9. House status

- Rent
- Ownership

10. Monthly income

- < 500 JD
- 500-1000 JD
- > 1000 JD

11. Number of family members

- Less than 4
- 4-7
- More than 7

12. Health insurance

- Yes
- No

Food Security status

**HFOOD 1**	Which of these sentences applies the most to the food eaten by your household during the past 6 months?	We had enough to eat of the kinds of food we wanted (quantity & quality)	We had enough to eat but not always the kinds of food we wanted (only quantity)	Sometimes we did not have enough to eat (quantity)	Often we did not have enough to eat	**Don’t Know/Refuse to answer**
			**Yes**	**No**	**Don’t Know/Refuse to answer**	
**HFOOD 2**	In the last 6 months, was there a time when you were concerned that you would run out of food for your household for the next month?					
**HFOOD 3**	Did the following statement apply to your household in the last 6 months? "The food that we bought was not enough and we didn't have money to get more."					
**HFOOD 4**	Are there any foods you feel your family does not eat enough of?					
**HFOOD 5**	In the past 6 months, did you or any other adult in your household ever cut the size of your meal because there was not enough food?					
**HFOOD 6**	In the past 6 months, did you or any other adult ever skip a meal because there was not enough food?					
**HFOOD 7**	In the past 6 months did you or any adult in your household not eat for a whole day or go to bed hungry because there was not enough food?					
**HFOOD 8**	During the last 6 months, was there a time when you or any adult in your household were unable to eat healthy and nutritious food because of a lack of money or other resources?					
**HFOOD 9**	During the last 6 months, was there a time when you or any adult in your household were hungry but did not eat because there was not enough money or other resources for food?					
**HFOOD 10**	During the last 6 months, was there a time when you or any adult in your household went without eating for a whole day because of a lack of money or other resources?					

Food Consumption and coping strategies

1. Yesterday, how many meals were eaten by this household? _______________ Meals
2. Is this number of meals:

   - Same as always

   - Less than usual

   - More than usual
3. Over the last 7 days, how many days did you consume the following foods? (No value can be greater than 7, i.e. 7 = 7 days)

   a) Cereals (bread, pasta, wheat flour, burghul)

   b) White tubers and roots (potato, sweet potato)

   c) Vegetables, yellow tubers, leaves

   d) Fruits

   e) Eggs

   f) Fish and other seafood

   g) Pulses, nuts and seeds (beans, chickpeas, etc.)

   h) Milk and dairy products

   i) Oil and fats

   j) Sweets (sugar, honey, jam, cakes, sweet coffee)

   k) Spices and condiments

   l) Meat
4. During the last 7 days, how many times (in days) did your household do any of the following in order to cope with lack of food? (No value can be greater than 7, i.e. 7=7 days; 0 = None, 1 = 1 day, 2 = 2 days, 3 = 3 days, 4 = 4 days, 5 = 5 days, 6 = 6 days, 7 = Everyday)

   a) Eat cheaper food that is not as good as normal

   b) Borrowed food or received help from friends or relatives

   c) Eaten less meals a day than normal

   d) Eaten smaller amounts of food than normal at meals

   e) Adults eat less so younger children can eat
5. During the last 12 months, was there a time when, because of lack of money or other resources:

a) You were worried you would not have enough food to eat?

b) You were unable to eat healthy and nutritious food?

c) You ate only a few kinds of foods?

d) You had to skip a meal?

e) You ate less than you thought you should?

f) Your household ran out of food?

g) You were hungry but did not eat?

h) You went without eating for a whole day?

6. In the past 30 days, has your household done any of the following to meet basic food needs? 0 = No, 1 = Yes, 2 = No, because I have already used this up

a) Spent savings

b) Bought food on credit or borrowed money to buy food

c) Asked for remittances

d) Spent less money on other needs (e.g. education/health)

e) Sold household assets (jewelry, phone, furniture, etc.)

f) Sold productive goods/assets (sewing machine, tools/machinery, car, livestock, etc.)

g) Taken jobs that are high risk, illegal and/or socially degrading

h) Any type of labor for food

k) Asked charity (local charity, religious institutions…) for food

l) Received E-cards from the UN

m) Other
